# Skin Autofluorescence and Subclinical Atherosclerosis in Mild to Moderate Chronic Kidney Disease: A Case-Control Study

**DOI:** 10.1371/journal.pone.0170778

**Published:** 2017-01-31

**Authors:** Enric Sánchez, Àngels Betriu, David Arroyo, Carolina López, Marta Hernández, Ferran Rius, Elvira Fernández, Albert Lecube

**Affiliations:** 1 Endocrinology and Nutrition Department. Hospital Universitari Arnau de Vilanova de Lleida, Institut de Recerca Biomèdica de Lleida (IRBLleida), Universitat de Lleida, Lleida, Catalonia, Spain; 2 Unit for the Detection and Treatment of Atherothrombotic Diseases (UDETMA), Nephrology Department, Hospital Universitari Arnau de Vilanova de Lleida, Institut de Recerca Biomèdica de Lleida (IRBLleida), Universitat de Lleida. Lleida, Catalonia, Spain; 3 CIBER de Diabetes y Enfermedades Metabólicas Asociadas (CIBERDEM), Instituto de Salud Carlos III (ISCIII), Madrid, Spain; Hospital Universitario de la Princesa, SPAIN

## Abstract

Advanced glycation end-products (AGEs) are increased and predict mortality in patients with chronic kidney disease (CKD) who are undergoing hemodialysis, irrespective of the presence of type 2 diabetes. However, little information exits about the relationship between AGEs and subclinical atherosclerosis at the early stages of CKD. A case-control study was performed including 87 patients with mild-to-moderate stages of CKD (glomerular filtration rate from 89 to 30 ml/min/per 1.73m^2^) and 87 non-diabetic non-CKD subjects matched by age, gender, body mass index, and waist circumference. Skin autofluorescence (AF), a non-invasive assessment of AGEs, was measured. The presence of atheromatous disease in carotid and femoral arteries was evaluated using vascular ultrasound, and vascular age and SCORE risk were estimated. Patients with mild-to-moderate stages of CKD showed an increase in skin AF compared with control subjects (2.5±0.6 *vs*. 2.2±0.4 AU, p<0.001). A skin AF value >2.0 AU was accompanied by a 3-fold increased risk of detecting the presence of an atheromathous plaque (OR 3.0, 95% CI 1.4–6.5, p = 0.006). When vascular age was assessed through skin AF, subjects with CKD were almost 12 years older than control subjects (70.3±25.5 *vs*. 58.5±20.2 years, p = 0.001). Skin AF was negatively correlated with glomerular filtration rate (r = -0.354, p<0.001) and LDL-cholesterol (r = -0.269, p = 0.001), and positively correlated with age (r = 0.472, p<0.001), pulse pressure (r = 0.238, p = 0.002), and SCORE risk (r = 0.451, p<0.001). A stepwise multivariate regression analysis showed that age and glomerular filtration rate independently predicted skin AF (R^2^ = 0.289, p<0.001). Skin AF is elevated in patients with mild-to-moderate CKD compared with control subjects. This finding may be independently associated with the glomerular filtration rate and the presence of subclinical atheromatous disease. Therefore, the use of skin AF may help to accurately evaluate the real cardiovascular risk at the early stages of CKD.

## Introduction

Advanced glycation end products (AGEs) characterize a heterogeneous group of compounds formed by the non-enzymatic glycation of proteins after exposure to aldose sugars [1]. These reactions progress in normal aging, and are accelerated under chronic hyperglycemia [2, 3]. In this way, the concentration of AGEs is associated with a higher incidence and faster progression of chronic type 2 diabetes (T2D) microangiopathy, and it is also an independent predictor of mortality in this population [4, 5]. In addition, other conditions like chronic inflammation, oxidative stress, and tobacco smoke can lead to increased AGES formation [1, 6, 7].

The gold standard skin biopsy measurement of AGEs agglomeration may be substituted by a non-invasive device based on skin autofluorescence (AF) [8]. Skin AF has been previously validated in clinical settings, and its clinical value has been established in large studies including individuals with a high risk of atherosclerosis, as T2D and chronic kidney disease (CKD) [9–12]. AGEs promote the development and evolution of atherosclerosis through direct and receptor pathways [13].

The progressive loss of glomerular filtration rate (GFR) is associated with systemic inflammation, as well as with an imbalance between oxygen reactive species production and antioxidant defenses [14, 15]. Increased circulating levels of AGEs are found in patients with CKD undergoing hemodialysis regardless, of the presence of T2D [4, 16]. Some additional factors have been associated with AGEs accumulation in renal failure because of decreased glomerular filtration, intraperitoneal formation during the time course of peritoneal dialysis, or dietary intake [17–20]. Therefore, the high body burden of AGEs in subjects with CKD may play a role in the pathogenesis of vascular complications associated with hemodialysis [21]. However, there is little information about the relationship between AGEs and subclinical atherosclerosis at earlier stages of CKD.

To shed light on this issue, we performed a case-control study of tissue accumulation of AGEs according to the presence of mild to moderate CKD. For this purpose, we selected subjects without T2D and no previous cardiovascular events. The AGEs were measured via skin AF. We also aimed to assess the relationship between AGEs accumulation and subclinical atheromatosis, by evaluating vascular ultrasound data.

## Material and Methods

### Ethics statement

Informed written consent was obtained from all participants, and the protocol was approved by the Arnau de Vilanova University Hospital ethics committee.

### Design of the study and description of the study population

We assessed the effect of mild to moderate CKD on tissue accumulation of AGEs following the *Strengthening the Reporting of Observational Studies in Epidemiology* guidelines for reporting case-control studies [22].

A total of 128 patients attending the outpatient Nephrology Clinic were examined to determine eligibility at the time of a regular visit between December 2014 and October 2015. The inclusion criteria were age older than 18 years, Caucasian origin, and GFR categories G2 (mildly decreased; 60–89 ml/min/per 1.73m^2^), G3a (mildly to moderately decreased; 45–59 ml/min/per 1.73m^2^), or G3b (moderately to severely decreased; 30–44 ml/min/per 1.73m^2^) according the standards established by the *Kidney Disease*: *Improving Global Outcomes* [23]. Therefore, all patients with GFR category G2 also present moderately or increased albuminuria (≥ 30 mg/g or ≥ 3 mg/mmol). The GFR was estimated following the CKD-EPI (*Chronic Kidney Disease Epidemiology Collaboration*) equation [24].

Using the standard deviation of serum AGEs detected in a previous study, we determined that the minimum sample required was 51 subjects [2]. Forty-one patients were excluded: T2D (n = 10), prior cardiovascular event (n = 8), GFR lower than 30 ml/min/per 1.73m^2^ (n = 6), non-Caucasian races (n = 4), chronic treatment with steroids (n = 3), active malignancy (n = 3), type 1 diabetes (n = 3), and age older than 80 years (n = 1). Moreover, 3 patients were excluded for their brown skin (Fitzpatrick type IV skin) because the excessive light absorption produced by this type of skin precludes reliable measurements of skin AF. No pregnant women were evaluated.

We aimed to select one control for every case. Subsequently, 87 subjects without kidney disease (GFR categories G1 and G2 without albuminuria) from the same Department served as the control group. Controls were closely matched to cases by, gender, BMI, waist circumference, and smoking status. As a linear relation between skin AF and subject age has been previously described, both groups were also matched by chronological age [2].

### Measurement of AGEs accumulation and determination of vascular age

Skin AF was measured using the AGE Reader™ device (DiagnOptics, Groningen, The Netherlands), a fully automated noninvasive tool that measures AGE deposition using an Ultraviolet-A spectrum. The skin AF is determined from the ratio between the emission fluorescence in the wavelength range between 420–600 nm, and the reflected excitation light with a wavelength between 300–420 nm, which was measured using a spectrometer and software. The measurement time is about one minute, and the mean value of three readings was recorded in all subjects. In addition, vascular age was calculated using skin AF value by the formula previously validated by Koetsier [vascular age = (skin AF– 0.83) / 0.024)] [2].

### Vascular ultrasound study and SCORE risk estimation

The ultrasound assessment of carotid and femoral arteries followed a predetermined protocol as defined in the NEFRONA study [25]. Briefly, B-mode and color-Doppler ultrasound imaging was performed using a Vivid-i BT09device (General Electrics Healthcare, Waukesha, WI) equipped with 6–13 MHz broadband linear array transducer and Doppler examinations in transverse and longitudinal planes. The presence of atheromatous plaque in the following territories was evaluated on the left and right sides: internal, bulb and common carotid arteries, and deep and superficial femoral arteries. Plaques were defined as focal intrusions into the lumen ≥1.5 mm thick, as recommended by *American Society of Echocardiography* [26]. Simultaneously, the ankle-brachial index (ABI) was assessed: a pathologic ABI was defined as a value ≤0.9 or ≥1.4, and the modified method by Schröeder was preferred [27]. Participants were classified by grades of atheromatous disease in 4 stages according ultrasound study and the ABI: **(i)** no atherosclerosis (ABI >0.9); **(ii)** mild atherosclerosis (ABI between 0.7–0.9); **(iii)** moderate atherosclerosis (carotid plaque with stenosis <50%); and **(iv)** severe atherosclerosis (ABI <0.7 or carotid plaque with stenosis ≥50%) [25]. To better analyze our results, patients were grouped according to the severity of atheromatous disease: Group I (patients without and with mild atherosclerosis, in which the absence of plaques is mandatory) and Group II (patients with moderate and severe atherosclerosis, in which presence of plaques is mandatory).

The SCORE (Systematic COronary Risk Evaluation) risk system is based on age, gender, country of origin, systolic blood pressure, smoking status, and either total cholesterol or total cholesterol/high-density lipoprotein cholesterol ratio. It was used to estimate the 10-year risk of mortality from cardiovascular disease [28].

### Statistical analysis

Normal distribution of the variables was evaluated using the Kolmogorov-Smirnov test. Data were expressed either as the mean ± SD or median (total range). Comparisons between groups were performed using the Student’s *t* test or the Mann-Whitney U test for continuous variables, and the χ^2^ test or the Fisher test were used for categorical variables.

The relationship between the continuous variables was examined with Pearson’s linear correlation test or the Spearman correlation coefficient. A stepwise multivariate regression analysis was used to explore the variables independently related to skin AF. The independent variables included age, gender, pulse pressure, LDL cholesterol, GFR, glycosylated haemoglobin, and SCORE risk. Significance was considered with a two-sided p value <0.05. Statistical analyses were performed using SSPS statistical package (SPSS, Chicago, IL, USA) version 20.

## Results

**Fig 1:**
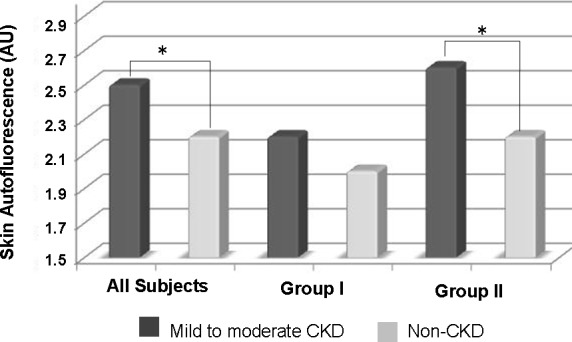
Skin autofluorescence according to the presence of atheromatous plaque between controls and patients with mild to moderate stages of CKD. CKD: chronic kidney disease; AU: arbitrary units; *: p<0.001; Group I: patients without and with mild atherosclerosis, in which the absence of plaques is mandatory; Group II: patients with moderate and severe atherosclerosis, in which presence of plaques is mandatory.

The main clinical characteristics and metabolic data of the study population according to the presence of CKD are showed in **Table 1**. Patients with mild to moderate decrease in GFR showed significantly higher levels of skin AF versus non-CKD subjects (2.5 ± 0.6 *vs*. 2.2 ± 0.4 arbitrary units (AU), p<0.001). When the subjects with and without atheromatous plaque (group I vs. group II) were analyzed separately, differences in skin AF values persisted only in the second group (Group II: 2.6 ± 0.5 *vs*. 2.2 ± 0.5 AU, p<0.001), and disappeared among subjects with no detectable plaque (Group I: 2.2 ± 0.7 *vs*. 2.0 ± 0.3 AU, p = 0.464) (**Fig 1**). When the entire population was evaluated, subjects with a skin AF value higher > 2.0 AU showed a 3-fold increased risk of an atheromathous plaque (OR 3.0, 95% CI 1.4–6.5, p = 0.006).

**Table 1:** Main clinical characteristics and metabolic data of the study population according to the presence of chronic kidney disease.

	*Mild to moderate CKD*	*Non CKD*	*Mean difference (95% CI)*	*p*
**N**	87	87	-	-
**Women, n (%)**	33 (37.9)	33 (37.9)	-	1.000
**Age (yrs)**	58.1 ± 10.6	56.5 ± 8.8	-1.5 (-4.4 to 1.4)	0.307
**BMI (Kg/m**^**2**^**)**	28.8 ± 5.8	28.9 ± 4.8	0.8 (-1.5 to 1.6)	0.918
**Waist circumference (cm)**	100.4 ± 15.1	100.7 ± 12.7	0.2 (-3.9 to 4.5)	0.893
**Non-smoker, n (%)**	56 (49.5)	49 (41.4)	-	0.286
**Systolic Blood Pressure (mm Hg)**	131.1 ± 16.8	128.0 ± 18.0	-3.0 (-8.2 to 2.1)	0.246
**Diastolic Blood Pressure (mm Hg)**	77.6 ± 10.0	75.7 ± 11.7	-1.9 (-5.1 to 1.3)	0.253
**Pulse Pressure (mm Hg)**	53.5 ± 14.3	52.3 ± 14.3	-1.1 (-5.4 to 3.1)	0.591
**Fasting plasma glucose (mmol/l)**	5.3 ± 0.5	5.3 ± 0.8	0.0 (-3.1 to 4.7)	0.685
**HbA1c (%)**	5.4 ± 0.3	5.4 ± 0.4	0.0 (-0.1 to 0.1)	0.933
**Serum Creatinine (mg/dL)**	1.32 ± 0.6	0.81 ± 0.1	-0.5 (-0.6 to -0.3)	<0.001
**GFR (mL/min per 1.73m**^**2**^**)**	60.8 ± 18.3	90.0 ± 9.3	29.1 (24.7 to 33.5)	<0.001
**ACR (mg/g)**	108.2 ± 191.0	5.3 ± 5.3	-102.9 (-150.7 to -55.1)	<0.001
**Total cholesterol (mg/dL)**	164.5 ± 35.6	194.9 ± 41.9	30.4 (-18.7 to 42.1)	<0.001
**HDL-cholesterol (mg/dL)**	50.7 ± 12.6	53.2 ± 12.2	2.42 (-1.4 to 6.2)	0.218
**LDL-cholesterol (mg/dL)**	91.4 ± 27.9	117.4 ± 37.2	26.0 (15.8 to 36.3)	<0.001
**Triglycerides (mg/dL)**	133.5 (42.0 to 780.0)	140.0 (52.0 to 632)	6.4 (-21.6 to 34.6)	0.650
**SCORE risk (%)**	2.3 ± 2.6	1.7 ± 2.2	-0.6 (-1.3 to 0.0)	0.079
**Atheromatous plaque, n (%)**	68 (78.1)	72 (82.7)	-	0.444
**Causes of chronic kidney disease**				
** High blood pressure**	36 (41.4)	-	-	-
** Polycystic kidney disease**	16 (18.4)	-	-	-
** Glomerulonephritis**	25 (28.7)	-	-	-
** Tubulointerstitial nephritis**	10 (11.5)	-	-	-

Data are means ± SD, n (percentage) or median (total range). CKD: chronic kidney disease; BMI: body mass index; HbA1c: glycosylated haemoglobin; GFR: glomerular filtration rate estimated according the CKD-EPI (*Chronic Kidney Disease Epidemiology Collaboration*) equation; ACR: albumin to creatinine ratio; SCORE: Systematic Coronary Risk Evaluation.

As shown in previous studies, a strong positive correlation was observed between skin AF and age (r = 0.472, p<0.001), without differences between genders. While both groups were closely matched for age, when vascular age was assessed, subjects with mild to moderate CKD appeared to be almost twelve years older than control subjects (70.3 ± 25.5 *vs*. 58.5 ± 20.2 years, p = 0.001).

**Fig 2:**
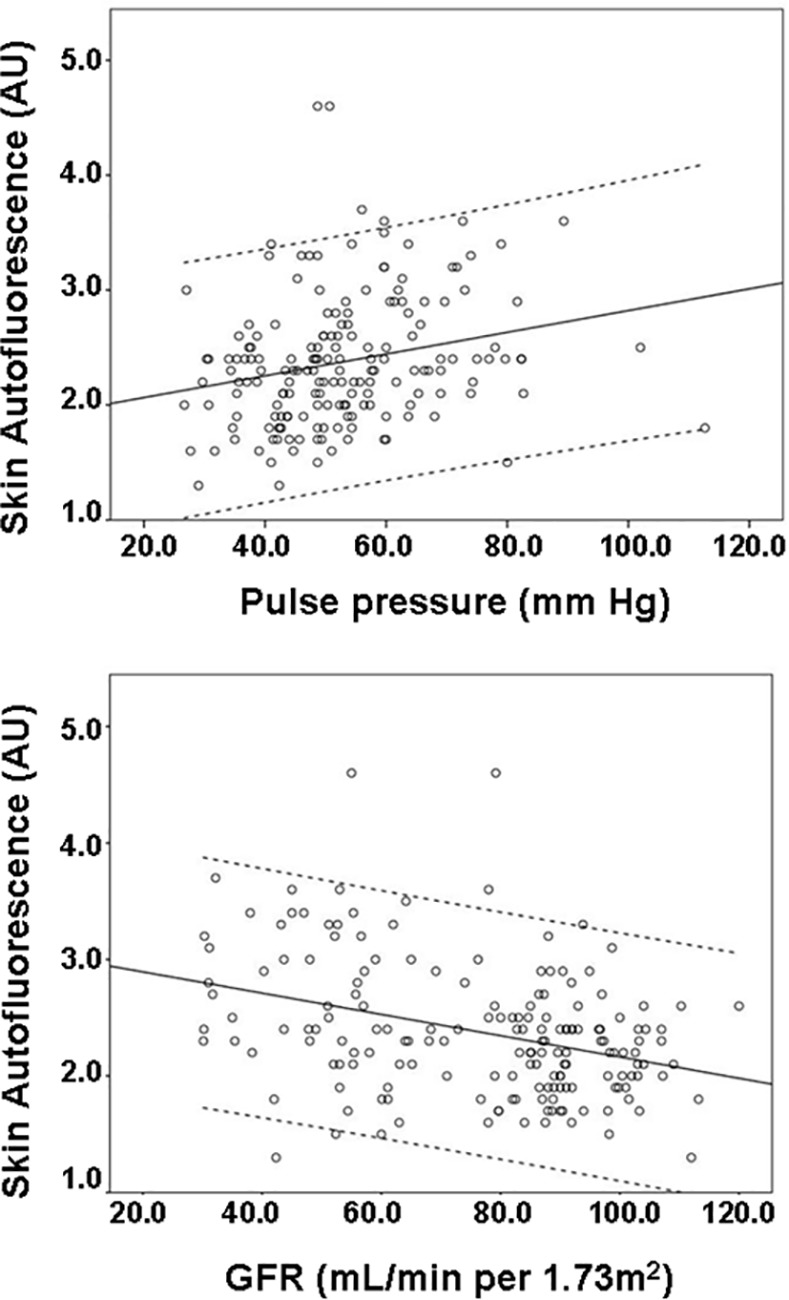
Scatter plot showing linear correlation between skin autofluorescence and: pulse pressure, and glomerular filtration rate. AU: arbitrary units; GFR: glomerular filtration rate estimated according the CKD-EPI (*Chronic Kidney Disease Epidemiology Collaboration*) equation.

In the entire population, skin AF correlated negatively with GFR (r = -0.354, p<0.001), and LDL-cholesterol (r = -0.269, p = 0.001), and correlated positively with age (r = 0.472, p<0.001), pulse pressure (r = 0.238, p = 0.002), and SCORE risk (r = 0.451, p<0.001). (**Fig 2**). The same linear correlations were observed when only patients with CKD were evaluated, but disappeared in the control group. An intriguing negative correlation was also stablished between AGEs and LDL cholesterol (**Table 2**).

**Table 2:** Correlations of skin autofluorescence with clinic and metabolic variables.

	*All subjects*		*Mild to moderate CKD*		*Non CKD*	
	*r*	*p*	*r*	*p*	*r*	*p*
**Age (yrs)**	0.472	<0.001	0.586	<0.001	0.291	0.006
**BMI (kg/m**^**2**^**)**	0.040	0.600	0.088	0.417	-0.25	0.819
**PP (mmHg)**	0.238	0.002	0.380	<0.001	0.055	0.612
**FPG (mmol/l)**	-0.021	0.789	0.016	0.885	-0.040	0.712
**HbA1c (%)**	0.054	0.603	0.139	0.307	-0.124	0.453
**GFR (mL/min per 1.73m**^**2**^**)**	-0.349	<0.001	-0.315	0.003	-0.110	0.309
**LDL-cholesterol (mg/dl)**	-0.269	0.001	-0.127	0.255	-0.284	0.011
**SCORE risk (%)**	0.451	<0.001	0.541	<0.001	0.314	0.105

CKD: chronic kidney disease; BMI: body mass index; PP: pulse pressure; FPG: fasting plasma glucose; HbA1c: glycosilated hemoglobin; GFR: glomerular filtration rate estimated according the CKD-EPI (*Chronic Kidney Disease Epidemiology Collaboration*) equation; SCORE: Systematic Coronary Risk Evaluation).

Finally, a stepwise multivariate regression analysis showed that the age and GFR (but not pulse pressure, glycosylated hemoglobin, LDL-cholesterol nor SCORE risk) were independently associated with forearm skin AF (R^2^ = 0.289, p<0.001) (**Table 3**).

**Table 3:** Stepwise multivariate regression analysis of variables associated with skin autofluorescence.

		Beta	p
**Skin AF**	**Age (yrs)**	0.424	<0.001
	**GFR (mL/min per 1.73m**^**2**^**)**	-0.275	<0.001
	**LDL-cholesterol (mg/dl)**	-0.148	0.112
	**SCORE risk (%)**	0.155	0.171
	**PP (mmHg)**	0.057	0.598
	**HbA1c (%)**	0.032	0.719
R^2^ = 0.289	*Constant*		0.001

Beta: Standardized regression coefficient; AF: autofluorescence; GFR: glomerular filtration rate estimated according the CKD-EPI (*Chronic Kidney Disease Epidemiology Collaboration*) equation; SCORE (Systematic Coronary Risk Evaluation); PP: pulse pressure; HbA1c: glycosilated haemoglobin.

## Discussion

To the best of our knowledge, this is the first study to show that subjects with early stages of CKD significantly increase skin AF values. In addition, a close relationship exists between skin AF and asymptomatic atheromatous disease in this population. Furthermore, skin AF appears to be negatively correlated with GFR, suggesting that renal dysfunction is a key factor to increase AGEs deposition in subcutaneous tissue.

The current study agrees with recent findings in subjects with an estimated GFR>60 ml/min/per 1.73m^2^, in whom skin AF was significantly higher in those with peripheral artery disease versus the subclinical atherosclerosis group. This contributes to vascular damage in addition to classical mechanisms [29]. The biological effects of AGEs through its ligation to their receptors located in large blood vessels accelerate plaque formation [13]. In patients with end-stage renal disease, immunostained pentosidine -a major glycoxidation product- was observed along the elastin fibers in aortic media. This was associated with medial calcification [30]. These data also shown that deposition of AGEs accompanies subclinical atherosclerosis beyond the presence of T2D [31].

CKD substantially increases the risk of cardiovascular disease. Indeed, a large community-based population study including 1,120,295 adults showed an adjusted hazard ratio for cardiovascular events was 1.4 with an estimated GFR of 45 to 59 ml/min/per 1.73m^2^. This increased to 2.0 with an estimated GFR of 30 to 44 ml/min/per 1.73m^2^ [32]. In addition, AGEs are also known to accumulate in the microvasculature of the kidney and to promote glomerular filtration and proteinuria [33]. In fact, clinical studies in patients with type 1 diabetes revealed a significant increase in the skin concentration of AGEs as urinary albumin increased from normal to microalbuminuria, and macroalbuminuria [34].

The plasma concentration of AGEs in T2D has also identified those normoalbuminuric subjects who will experience a higher increase in the glomerular basement membrane at in about 5-years follow-up period [35]. More recently, Luo et al. have demonstrated that in a non-hyperglycemic milieu, AGEs increase the permeability of the glomerular endothelial cells by a matrix metalloproteinases degradation of tight junction complexes, mainly occluding and claudin-5 proteins [36]. Our results support the close relationship between AGEs accumulation and decreases in GFR because a strong and negative relationship between mild to moderate ranges of GFR and skin AF was observed. Whether the AGEs renal accumulation in humans promotes kidney dysfunction or whether the decrease in GRF triggers AGE accumulation cannot be elucidated from our study. However, when the receptor for AGEs is deleted in a mouse model, there is a 29% increase in GFR accompanied by structural changes such as reduced thickening of glomerular basement membrane and mesangial sclerosis [37].

When assessed using skin AF values, we observed a marked increase in vascular age, which is more than 10 years higher than the chronological age in patients with CKD. Vascular aging occurs along with endothelial dysfunction, vascular remodelling, inflammation, and increased stiffness, all of them previously associated with AGEs [16, 38]. In this way, we observed a 3-fold increased risk of an atheromathous plaque in subjects with a skin AF value higher > 2.0 AU. This data support the idea that AGEs are useful in identifying a subclinical phenotype of early vascular disease in large blood vessels [39]. Therefore, in a CKD population before end-stage disease is established, skin AF may represent a clinically helpful and non-invasive method to screen assess cardiovascular risk.

The relationship between skin AF and other conventional risk factors outside T2D remains controversial. In our study, skin AF positively correlated with SCORE risk when the entire population as well as patients with renal impairment were evaluated. However, the correlation disappeared in the control group. Similarly, skin AF was not related to SCORE risk or its components in a sub-study of the *Groningen Overweight and Lifestyle* (GOAL) project that included overweight and obese subjects without T2D nor renal disease [40]. These data support the idea that, in the clinical setting the decreased GFR is as a key factor accounting for skin AF when T2D is not present. The inverse association between skin AF and LDL cholesterol detected in our population deserves an additional comment. When the LDL conjugated diene is measured as marker of lipid peroxidative stress, a negative correlation with skin AF has been described in critically ill patients [41]. In addition, serum LDL cholesterol was also negatively correlated with skin AF in a cross-sectional study of 223 individuals visiting the vascular outpatient clinic for primary or secondary prevention [29].

This study has some limitations. As a cross-sectional study, we cannot establish a causal relationship between skin AF and subclinical atheromatosis. However, the problem is clinically relevant since the prevalence of CKD reaches 20.4% among participants from the 2005–2006 *National Health and Nutrition Examination Survey* (NHANES), and help is needed to better identify subjects at risk [42, 43]. Second, we did not compared skin AF with plasma AGEs levels. We assumed that skin AF remains stable for a long time because it is less influenced by factors such as smoking or nutrition. In fact, plasma AGEs measurements were not different when comparing individual with and without cardiovascular disease in participants from two Dutch cohort studies including 1.291 subjects with various degrees of glucose metabolism [44]. Third, skin AF could be unreliable in subjects with dark skin due to excessive light absorption. We tried to solve this limitation selecting only Caucasian subjects and excluding four of them with medium brown skin.

In conclusion, skin AF is elevated in patients with mild to moderate CKD in comparison with control subjects. This finding is related with the presence of subclinical atheromatous disease, and appears to be independently associated with the GFR. Therefore, skin AF is an easy, fast and non-invasive method that may help to accurately evaluate real cardiovascular risk in the early stages of CKD.

## References

[1] Schmidt AM, Hori O, Brett J, Yan SD, Wautier JL, Stern D. Cellular receptors for advanced glycation end products. Implications for induction of oxidant stress and cellular dysfunction in the pathogenesis of vascular lesions. Arterioscler Thromb. 1994;14: 1521–8. 7918300 10.1161/01.atv.14.10.1521

[2] Koetsier M, Lutgers HL, de Jonge C, Links TP, Smit AJ, Graaff R. Reference values of skin autofluorescence. Diabetes Technol Ther. 2010; 12: 399–403. 10.1089/dia.2009.0113 20388050

[3] **-**Vlassara H, Palace MR. Diabetes and advanced glycation end-products. J Intern Med. 2002; 251: 87–101. 11905595 10.1046/j.1365-2796.2002.00932.x

[4] Chilelli NC, Burlina S, Lapolla A. AGEs, rather than hyperglycemia, are responsible for microvascular complications in diabetes: a "glycoxidation-centric" point of view. Nutr Metab Cardiovasc Dis. 2013; 23: 913–9. 10.1016/j.numecd.2013.04.004 23786818

[5] Genuth S, Sun W, Cleary P, Sell DR, Dahms W, Malone J, Sivitz W, Monnier VM; DCCT Skin Collagen Ancillary Study Group. Glycation and carboxymethyllysine levels in skin collagen predict the risk of future 10-year progression of diabetic retinopathy and nephropathy in the Diabetes Control and Complications Trial and Epidemiology of Diabetes Interventions and Complications participants with type 1 diabetes. Diabetes 2005; 54: 3103–11. 16249432 10.2337/diabetes.54.11.3103 PMC2622724

[6] Yamagishi S, Matsui T. Advanced glycation end products, oxidative stress and diabetic nephropathy. Oxid Med Cell Longev. 2010; 3: 101–8. 10.4161/oxim.3.2.11148 20716934 PMC2952094

[7] Cerami C, Founds H, Nicholl I, Mitsuhashi T, Giordano D, Vanpatten S, Lee A, Al-Abed Y, Vlassara H, Bucala R, Cerami A. Tobacco smoke is a source of toxic reactive glycation products. Proc Natl Acad Sci USA. 1997; 94: 13915–20. 9391127 10.1073/pnas.94.25.13915 PMC28407

[8] Meerwaldt R, Graaff R, Oomen PH, Links TP, Jager JJ, Alderson NL, Thorpe SR, Baynes JW, Gans RO, Smit AJ. Simple non-invasive assessment of advanced glycation endproduct accumulation. Diabetologia 2004; 47: 1324–30. 10.1007/s00125-004-1451-2 15243705

[9] Lutgers HL, Graaff R, Links TP, Ubink-Veltmaat LJ, Bilo HJ, Gans RO, Smit AJ. Skin autofluorescence as a noninvasive marker of vascular damage in patients with type 2 diabetes. Diabetes Care. 2006; 29: 2654–9. 10.2337/dc05-2173 17130200

[10] Ueno H, Koyama H, Tanaka S, Fukumoto S, Shinohara K, Shoji T, Emoto M, Tahara H, Kakiya R, Tabata T, Miyata T, Nishizawa Y. Skin autofluorescence, a marker for advanced glycation end product accumulation, is associated with arterial stiffness in patients with end-stage renal disease. Metabolism 2008; 57: 1452–7. 10.1016/j.metabol.2008.05.016 18803952

[11] Meerwaldt R, Hartog JW, Graaff R, Huisman RJ, Links TP, den Hollander NC, Thorpe SR, Baynes JW, Navis G, Gans RO, Smit AJ. Skin autofluorescence, a measure of cumulative metabolic stress and advanced glycation end products, predicts mortality in hemodialysis patients. J Am Soc Nephrol. 2005; 16: 3687–93. 10.1681/ASN.2005020144 16280473

[12] Yamagishi S, Fukami K, Matsui T. Evaluation of tissue accumulation levels of advanced glycation end products by skin autofluorescence: A novel marker of vascular complications in high-risk patients for cardiovascular disease. Int J Cardiol. 2015; 185: 263–8. 10.1016/j.ijcard.2015.03.167 25814214

[13] Jandeleit-Dahm K, Cooper ME. The role of AGEs in cardiovascular disease. Curr Pharm Des 2008; 14: 979–86. 18473849 10.2174/138161208784139684

[14] Li L, Astor BC, Lewis J, Hu B, Appel LJ, Lipkowitz MS, Toto RD, Wang X, Wright JT Jr, Greene TH. Longitudinal progression trajectory of GFR among patients with CKD. Am J Kidney Dis. 2012; 59: 504–12. 10.1053/j.ajkd.2011.12.009 22284441 PMC3312980

[15] Poulianiti KP, Kaltsatou A, Mitrou GI, Jamurtas AZ, Koutedakis Y, Maridaki M, Stefanidis I, Sakkas GK, Karatzaferi C. Systemic redox imbalance in chronic kidney disease: a systematic review. Oxid Med Cell Longev. 2016; 2016: 8598253 10.1155/2016/8598253 27563376 PMC4987477

[16] Wang CC, Wang YC, Wang GJ, Shen MY, Chang YL, Liou SY, Chen HC, Chang CT. Skin Autofluorescence Is Associated with Endothelial Dysfunction in Uremic Subjects on Hemodialysis. PLoS One. 2016; 11: e0147771 10.1371/journal.pone.0147771 26809145 PMC4726548

[17] Raj DSC, Choudhury D, Welbourne TC, Levi M. Advanced glycation end products: a nephrologist’s perspective. Am J Kid Dis. 2000; 35: 365–80. 10692262 10.1016/s0272-6386(00)70189-2

[18] Friedlander M, Wu Y, Elgawish A and Monnier V. Early and advanced glycosylation end products. Kinetics of formation and clearance in peritoneal dialysis. J Clin Invest. 1996; 97: 728–35. 10.1172/JCI118471 8609229 PMC507110

[19] Miyata T, Ueda Y, Shinzato T, Iida Y, Tanaka S, Kurokawa K, van Ypersele de Strihou C, Maeda K. Accumulation of albumin-linked and free-form pentosidine in the circulation of uremic patients with end-stage renal failure: renal implications in the pathophysiology of pentosidine. J Am Soc Nephrol. 1996; 7: 1198–206. 8866413 10.1681/ASN.V781198

[20] Uribarri J, Peppa M, Cai W, Goldberg T, Lu M, He C, Vlassara H. Restriction of dietary glycotoxins reduces excessive advanced glycation end products in renal failure patients. J Am Soc Nephrol. 2003; 14: 728–31. 12595509 10.1097/01.asn.0000051593.41395.b9

[21] Weiner DE, Tighiouart H, Amin MG, Stark PC, MacLeod B, Griffith JL, Salem DN, Levey AS, Sarnak MJ. Chronic kidney disease as a risk factor for cardiovascular disease and all-cause mortality: a pooled analysis of community-based studies. J Am Soc Nephrol. 2004; 15: 1307–1315. 15100371 10.1097/01.asn.0000123691.46138.e2

[22] von Elm E, Altman DG, Egger M, Pocock SJ, Gøtzsche PC, Vandenbroucke JP; STROBE Initiative. The Strengthening the Reporting of Observational Studies in Epidemiology (STROBE) statement: guidelines for reporting observational studies. Lancet. 2007; 370: 1453–7. 10.1016/S0140-6736(07)61602-X 18064739

[23] Stevens PE, Levin A; Kidney Disease: Improving Global Outcomes Chronic Kidney Disease Guideline Development Work Group Members. Evaluation and management of chronic kidney disease: synopsis of the kidney disease: improving global outcomes 2012 clinical practice guideline. Ann Intern Med. 2013; 158: 825–30. 10.7326/0003-4819-158-11-201306040-00007 23732715

[24] Levey AS, Stevens LA, Schmid CH, Zhang YL, Castro AF 3rd, Feldman HI, Kusek JW, Eggers P, Van Lente F, Greene T, Coresh J; CKD-EPI (Chronic Kidney Disease Epidemiology Collaboration). A New Equation to Estimate Glomerular Filtration Rate. Ann Intern Med. 2009 5 5;150(9):604–612. 19414839 10.7326/0003-4819-150-9-200905050-00006 PMC2763564

[25] Junyent M, Martínez M, Borràs M, Coll B, Valdivielso JM, Vidal T, Sarró F, Roig J, Craver L, Fernández E. Predicting cardiovascular disease morbidity and mortality in chronic kidney disease in Spain. The rationale and design of NEFRONA: a prospective, multicenter, observational cohort study. BMC Nephrol. 2010; 11: 14 10.1186/1471-2369-11-14 20609210 PMC2919528

[26] Stein JH, Korcarz CE, Hurst RT, Lonn E, Kendall CB, Mohler ER, Najjar SS, Rembold CM, Post WS; American Society of Echocardiography Carotid Intima-Media Thickness Task Force. Use of carotid ultrasound to identify subclinical vascular disease and evaluate cardiovascular disease risk: a Consensus Statement from the American Society of Echocardiography Carotid Intima-Media Thickness Task Force Endorsed by the Society for Vascular Medicine. J Am Soc Echocardiogr. 2008; 21: 93–111. 10.1016/j.echo.2007.11.011 18261694

[27] Schröder F, Diehm N, Kareem S, Ames M, Pira A, Zwettler U, Lawall H, Diehm C. A modified calculation of ankle-brachial pressure index is far more sensitive in the detection of peripheral arterial disease. J Vasc Surg. 2006: 44: 531–6. 10.1016/j.jvs.2006.05.016 16950430

[28] Conroy RM, Pyörälä K, Fitzgerald AP, Sans S, Menotti A, De Backer G, De Bacquer D, Ducimetière P, Jousilahti P, Keil U, Njølstad I, Oganov RG, Thomsen T, Tunstall-Pedoe H, Tverdal A, Wedel H, Whincup P, Wilhelmsen L, Graham IM; SCORE project group. Estimation of ten-year risk of fatal cardiovascular disease in Europe: the SCORE project. Eur Heart J. 2003; 24: 987–1003. 12788299 10.1016/s0195-668x(03)00114-3

[29] den Dekker MA, Zwiers M, van den Heuvel ER, de Vos LC, Smit AJ, Zeebregts CJ, Oudkerk M, Vliegenthart R, Lefrandt JD, Mulder DJ. Skin autofluorescence, a non-invasive marker for AGE accumulation, is associated with the degree of atherosclerosis. PLoS One. 2013; 8: e83084 10.1371/journal.pone.0083084 24376641 PMC3871581

[30] Sakata N, Noma A, Yamamoto Y, Okamoto K, Meng J, Takebayashi S, Nagai R, Horiuchi S. Modification of elastin by pentosidine is associated with the calcification of aortic media in patients with end-stage renal disease. Nephrol Dial Transplant. 2003; 18: 1601–9. 12897101 10.1093/ndt/gfg200

[31] Lutgers HL, Graaff R, de Vries R, Smit AJ, Dullaart RP. Carotid artery intima media thickness associates with skin autofluoresence in non-diabetic subjects without clinically manifest cardiovascular disease. Eur J Clin Invest 2010; 40: 812–7. 10.1111/j.1365-2362.2010.02329.x 20597962

[32] Go AS, Chertow GM, Fan D, McCulloch CE, Hsu CY. Chronic kidney disease and the risks of death, cardiovascular events, and hospitalization. N Engl J Med. 2004; 351: 1296–305. 10.1056/NEJMoa041031 15385656

[33] Rojas A, Morales MA. Advanced glycation and endothelial functions: a link towards vascular complications in diabetes. Life Sci. 2004; 76: 715–30. 10.1016/j.lfs.2004.09.011 15581904

[34] Beisswenger PJ, Makita Z, Curphey TJ, Moore LL, Jean S, Brinck-Johnsen T, Bucala R, Vlassara H. Formation of immunochemical advanced glycosylation end products precedes and correlates with early manifestations of renal and retinal disease in diabetes. Diabetes. 1995; 44: 824–9. 7789650 10.2337/diab.44.7.824

[35] Beisswenger PJ, Howell SK, Russell GB, Miller ME, Rich SS, Mauer M. Early progression of diabetic nephropathy correlates with methylglyoxal-derived advanced glycation end products. Diabetes Care. 2013; 36: 3234–9. 10.2337/dc12-2689 23780945 PMC3781566

[36] Luo P, Peng H, Li C, Ye Z, Tang H, Tang Y, Chen C, Lou T. Advanced glycation end products induce glomerular endothelial cell hyperpermeability by upregulating matrix metalloproteinase activity. Mol Med Rep. 2015; 11: 4447–53. 10.3892/mmr.2015.3269 25634678

[37] Reiniger N, Lau K, McCalla D, Eby B, Cheng B, Lu Y, Qu W, Quadri N, Ananthakrishnan R, Furmansky M, Rosario R, Song F, Rai V, Weinberg A, Friedman R, Ramasamy R, D'Agati V, Schmidt AM. Deletion of the receptor for advanced glycation end products reduces glomerulosclerosis and preserves renal function in the diabetic OVE26 mouse. Diabetes. 2010; 59: 2043–54. 10.2337/db09-1766 20627935 PMC2911065

[38] Kajikawa M, Nakashima A, Fujimura N, Maruhashi T, Iwamoto Y, Iwamoto A, Matsumoto T, Oda N, Hidaka T, Kihara Y, Chayama K, Goto C, Aibara Y, Noma K, Takeuchi M, Matsui T, Yamagishi S, Higashi Y. Ratio of serum levels of AGEs to soluble form of RAGE is a predictor of endothelial function. Diabetes Care. 2015; 38: 119–25. 10.2337/dc14-1435 25336748

[39] Harvey A, Montezano AC, Touyz RM. Vascular biology of ageing-Implications in hypertension. J Mol Cell Cardiol. 2015; 83: 112–21. 10.1016/j.yjmcc.2015.04.011 25896391 PMC4534766

[40] Tiessen AH, Jager W, ter Bogt NC, Beltman FW, van der Meer K, Broer J, Smit AJ. Skin autofluorescence as proxy of tissue AGE accumulation is dissociated from SCORE cardiovascular risk score, and remains so after 3 years. Clin Chem Lab Med. 2014; 52: 121–7. 10.1515/cclm-2012-0825 23612547

[41] Hunt KJ, Baker N, Cleary P, Backlund JY, Lyons T, Jenkins A, Virella G, Lopes-Virella MF; DCCT/EDIC Research Group. Oxidized LDL and AGE-LDL in circulating immune complexes strongly predict progression of carotid artery IMT in type 1 diabetes. Atherosclerosis. 2013; 231: 315–22. 10.1016/j.atherosclerosis.2013.09.027 24267245 PMC3924569

[42] Murphy D, McCulloch CE, Lin F, Banerjee T, Bragg-Gresham JL, Eberhardt MS, Morgenstern H, Pavkov ME, Saran R, Powe NR, Hsu CY; Centers for Disease Control and Prevention Chronic Kidney Disease Surveillance Team. Trends in Prevalence of Chronic Kidney Disease in the United States. Ann Intern Med. 2016 [Epub ahead of print] 10.7326/M16-0273 PMC5552458 27479614

[43] Shahinian VB, Hedgeman E, Gillespie BW, Young EW, Robinson B, Hsu CY, Plantinga LC, Burrows NR, Eggers P, Saydah S, Powe NR, Saran R; CDC CKD Surveillance System. Estimating prevalence of CKD stages 3–5 using health system data. Am J Kidney Dis. 2013; 61: 930–8. 10.1053/j.ajkd.2013.01.018 23489675 PMC4605141

[44] Hanssen NM, Engelen L, Ferreira I, Scheijen JL, Huijberts MS, van Greevenbroek MM. Plasma levels of advanced glycation endproducts Nε-(carboxymethyl)lysine, Nε-(carboxyethyl)lysine, and pentosidine are not independently associated with cardiovascular disease in individuals with or without type 2 diabetes: the Hoorn and CODAM studies. J Clin Endocrinol Metab. 2013; 98: E1369–73. 10.1210/jc.2013-1068 23780372

